# Emergent Bedside Thoracotomy for Decompression of an Incarcerated Neonatal Bochdalek Hernia: A Case Report

**DOI:** 10.1155/crpe/8858530

**Published:** 2026-02-13

**Authors:** Julian Kolorz, Christopher Lindow, Tomasz Baranski, Martin May, Britta Hüning, Johanna Bialas, Anja Stein, Ursula Felderhoff-Müser, Michael Berger, Jan Sabo

**Affiliations:** ^1^ Department of Pediatric Surgery, University Hospital Essen, University of Duisburg-Essen, Essen, Germany, uni-due.de; ^2^ Department of Pediatric Surgery, Elisabeth Hospital Essen, Essen, Germany, elisabeth-essen.de; ^3^ Department of Pediatrics I, University Hospital Essen, University of Duisburg-Essen, Essen, Germany, uni-due.de; ^4^ Center for Translational Neuro- and Behavioral Sciences (C-TNBS), University Hospital Essen, University of Duisburg-Essen, Essen, Germany, uni-due.de

**Keywords:** Bochdalek hernia, congenital diaphragmatic hernia (CDH), neonatal bedside thoracotomy

## Abstract

Congenital diaphragmatic hernia (CDH) is a rare and potentially life‐threatening condition caused by incomplete diaphragm formation, allowing abdominal organs to herniate into the thoracic cavity. This typically results in pulmonary hypoplasia and, rarely, cardiac compression with subsequent cardiopulmonary collapse. We report the emergency management of a three‐week‐old term neonate with a previously undiagnosed left‐sided Bochdalek hernia who presented at an external hospital with cardiac arrest and mediastinal shift due to massive obstructing enterothorax. Following stabilization and transfer to our hospital, a second cardiac arrest occurred in our neonatal intensive care unit (NICU). During cardiopulmonary resuscitation (CPR), an emergent bedside thoracotomy was performed, decompressing the thoracic cavity by exposing incarcerated bowel to the atmosphere, which led to the return of spontaneous circulation. Subsequent laparotomy revealed a small left‐sided diaphragmatic defect with incarcerated bowel. CDH repair and temporary abdominal wall closure using a silo patch were performed. Secondary closure was achieved 5 days later. The patient was discharged without complications. This case highlights emergent bedside thoracotomy as a life‐saving intervention in critical neonatal CDH.

## 1. Introduction

Congenital diaphragmatic hernia (CDH) is a rare developmental anomaly occurring in approximately 2.3 per 10,000 live births. It typically results from incomplete formation of the diaphragm, most commonly posterolaterally (Bochdalek hernia), allowing abdominal contents to herniate into the thoracic cavity [1–4]. This can impair lung development, leading to varying degrees of pulmonary hypoplasia and pulmonary hypertension [5]. In rare cases, CDH can cause progressive cardiopulmonary compromise, particularly when tension develops and herniated viscera lead to a mass effect, resulting in mediastinal shift, impaired venous return, and shock from cardiac compression. CDH typically presents within hours of birth with respiratory distress; however, late presentations, though rare, may be overlooked initially but are potentially life‐threatening due to nonspecific symptoms [6–9].

Thoracotomy in children is a well‐established emergency procedure in trauma settings as well as an acknowledged approach for elective and urgent repair for CDH, including in the NICU [10, 11]. To our best knowledge, however, there is no published literature for an emergent bedside thoracotomy in the NICU for a neonate with CDH and mass effect causing cardiopulmonary collapse in which decompression of heart, and lung was achieved by transposing the abdominal viscera into the atmosphere.

## 2. Case Report

**FIGURE 1:**
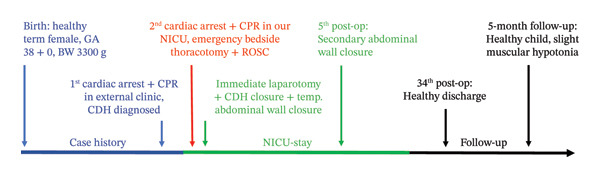
Timeline of events from birth to follow‐up, highlighting diagnosis, emergency bedside thoracotomy following cardiac arrest, and staged abdominal wall closure.

**FIGURE 2:**
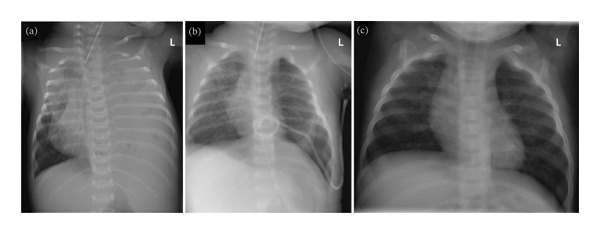
Initial chest radiograph at referring hospital showing a massive left diaphragmatic hernia with herniated bowel loops and rightward mediastinal shift (a). Immediate postoperative image after emergency bedside thoracotomy reveals re‐expansion of the left lung and resolution of mediastinal shift (b). Five‐month follow‐up radiograph shows normal lung aeration and thoracic anatomy without signs of recurrence (c).

A three‐week‐old female term neonate (gestational age 38 + 0 weeks, Apgar score 5′ 8, 10′ 9, birth weight 3300 g, weight at admission 3500 g) was admitted to our hospital after experiencing a cardiac arrest and successful CPR at an outside hospital due to a previously undiagnosed left‐sided diaphragmatic hernia (Bochdalek‐type) (Figure 1). The child had been previously healthy and had been discharged after birth without symptoms. Both prenatal screenings and postnatal clinical exams showed no signs suggestive of a CDH. The initial presentation was due to sudden onset of respiratory distress and lividity. A chest x‐ray performed at the external hospital showed a massive left‐sided enterothorax with mediastinal shift and cardiac compression (Figure 2(a)). Following successful CPR, the child was transferred to our hospital on inotropic support (dobutamine) and mechanical ventilation.

**FIGURE 3:**
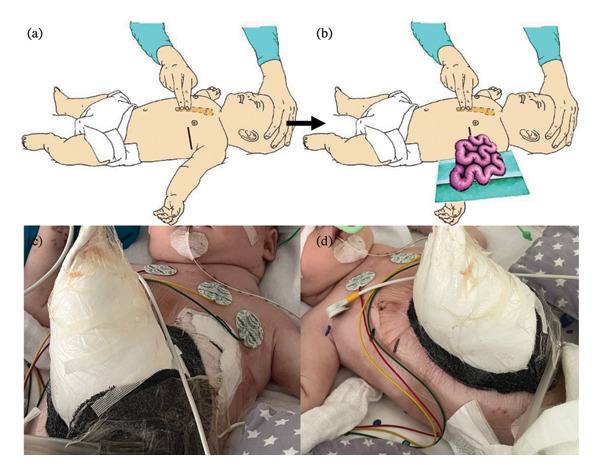
Schematic illustration of emergent bedside thoracotomy (a, b). Staged abdominal wall management following thoracic decompression, with visible thoracotomy site on the left. Placement of a silo for temporary coverage of the abdominal contents due to abdominal compartment concerns post‐thoracotomy (c, d).

After being transferred for corrective surgery, an echocardiogram showed compression of the heart towards the right side caused by the incarcerated left‐sided diaphragmatic hernia, resulting in severely reduced cardiac filling, myocardial contractility, and impaired output. Shortly afterwards, a second cardiac arrest occurred in our NICU. Immediate CPR was initiated again. Suspecting cardiac compression from tension enterothorax, a left‐sided bedside emergency thoracotomy was performed under semisterile conditions in the 5^th^ intercostal space using a scalpel and a clamp, gaining access to the thoracic cavity, and with the two index fingers bluntly spreading the ribs apart. Herniated small bowel loops were identified in the thoracic cavity, gently extracted, and temporarily exteriorized (Figures 3(a) and 3(b)). Within 2 minutes of decompression, spontaneous circulation was restored through ongoing vasopressor support, including dobutamine and norepinephrine.

Following stabilization, a sterile field was created, and the child’s abdomen was opened via a transverse subcostal laparotomy for definitive surgical repair. Intraoperative findings included a small (1.5 cm) posterolateral diaphragmatic defect on the left side, consistent with a Bochdalek hernia, and incarceration of edematous small bowel. Given the severely swollen bowel in reference to the small size of the hernia, for reducing the bowel first from the atmosphere into the thoracic cavity, and subsequently into the abdomen, the hernia needed to be opened for an additional 2 cm. The diaphragmatic defect was closed primarily using nonabsorbable sutures (Ethibond 2.0). In addition, a type II intestinal malrotation with right‐sided cecum and Ladd bands was diagnosed and corrected by dividing the bands and broadening the mesenteric trunk. Due to massive small bowel edema and concerns of abdominal compartment syndrome, the abdominal cavity could not be closed primarily. A Gore‐Tex patch was sutured in place to form a temporary silo (Figures 3(c) and 3(d)). No ischemia or necrosis was noted. A tunneled thoracic pigtail drain was inserted into the left thoracic cavity and a circular vacuum dressing was applied around the fascia‐patch suture line, sealed with VAC film to ensure effective drainage of ascites, and set to −50 mmHg suction (Figure 2(b)).

On postoperative day 5, during the planned secondary abdominal wall closure, four incipient small bowel perforations were noted and closed using single‐layer seromuscular interrupted sutures, without the necessity for bowel resection. The abdominal wall was closed, and a new chest tube was placed due to persistent pleural effusion.

The transient left‐sided chylothorax resolved under an MCT‐based, fat‐restricted enteral diet administered for 10 days, with subsequent gradual transition to standard feeding. Intraoperatively and immediately afterwards, the patient developed significant anemia and coagulopathy consistent with disseminated intravascular coagulation (DIC), necessitating urgent transfusion of multiple blood products to stabilize hemostasis and circulatory function.

The patient was mechanically ventilated for a total of 8 days, including a brief period of high‐frequency oscillatory ventilation (HFO) immediately after thoracic decompression. Following cardiopulmonary stabilization and extubation, she required noninvasive respiratory support via high‐flow nasal cannula and CPAP for another week. Oxygen supplementation was discontinued after that time, and spontaneous breathing remained stable thereafter.

Cranial MRI revealed bilateral Grade II intraventricular hemorrhage (IVH) (left > right), without evidence of parenchymal injury or hydrocephalus. Neurological function remained without symptoms throughout hospitalization.

Enteral feeding was advanced stepwise, complicated by mild gastric dysmotility and delayed stool passage, but was ultimately successful. A rhinovirus infection and minor Staphylococcus aureus skin colonization were managed supportively.

The patient was discharged after 34 days without neurological, pulmonary, cardiological, or nutritional impairments. At the 5‐month follow‐up, the child demonstrated physiological development and stable weight gain; a mild muscular hypotonia and only sporadic fidgety movements (indicating an increased risk for the development of cerebral palsy) were noted on the neurological examination. A follow‐up chest radiograph showed normal lung aeration and no signs of diaphragmatic recurrence or thoracic deformity (Figure 2(c)).

## 3. Discussion

CDH remains a complex condition with wide‐ranging presentations and variable outcomes. While the majority of CDH cases are identified prenatally or shortly after delivery due to respiratory distress, late‐presenting forms are less predictable. They can present with subtle or acute symptoms [6–9]. In our case, the child was asymptomatic after birth and was discharged home in good health, underscoring the diagnostic challenge posed by occult forms of CDH.

To our knowledge, there are only a few reports in the literature describing bedside thoracotomy in neonates for nontraumatic causes. Most available literature focuses on emergency thoracotomy in pediatric trauma [10, 11]. Allen et al. conducted a large case series and systematic review of pediatric emergency department thoracotomies and found that, despite frequent application in traumatic settings, survival rates remained exceedingly low, especially in younger patients [10]. Similarly, Glassman and Burjonrappa analyzed data from the National Trauma Data Bank and confirmed that emergency thoracotomy in children is an extremely rare intervention, largely limited to cases of penetrating trauma, with poor overall outcomes [11]. These findings underscore that emergency thoracotomy in children is generally viewed as a last‐resort intervention, applied only when all other options are exhausted and time is critical.

In our case, the first cardiac arrest occurred at the referring hospital and was successfully reversed with basic resuscitative efforts. The second arrest, however, occurred during preoperative preparations in our NICU. Initial echocardiography demonstrated severe rightward displacement and compression of the heart with minimal filling and contractility, hallmarks of obstructive physiology. The decision to perform an emergent thoracotomy at the bedside during CPR was based on these imaging findings, the absence of pneumothorax, and clinical deterioration. Serial echocardiograms demonstrated no patent ductus arteriosus (PDA). A left‐to‐right shunt across the foramen ovale was present, which is physiologic in neonates and not hemodynamically relevant in this case. The severely reduced cardiac filling was attributed to reversible mediastinal compression. Comparable interactions between diaphragmatic herniation and altered neonatal cardiac hemodynamics have been described in the literature, underscoring the importance of thorough echocardiographic evaluation [12].

Thoracic decompression via thoracotomy allowed immediate pressure release, exposing the herniated bowel loops to the atmosphere and thereby restoring venous return. In our retrospective analysis, compared to laparotomy, this was the pivotal decision which ultimately made this case successful. Laparotomy would have required a significantly more time‐consuming repositioning of the compressing, herniated bowel. As mentioned above, the hernia was small, and reduction of the abdominal visceral back into the abdomen required clear identification of the anatomy with subsequent widening of the hernia. Given the massively swollen bowel, this would have been both time‐consuming and cumbersome with the child undergoing active CPR. Therefore, in our analysis, thoracotomy unequivocally provided faster and more effective relief, since this approach was instrumental in achieving return of spontaneous circulation within minutes.

Surgical findings revealed not only a Bochdalek‐type hernia but also an associated intestinal malrotation. The inability to close the abdomen due to bowel distension required temporary silo placement. During second‐look surgery, incipient small bowel perforations were discovered and repaired without the need for resection. These injuries were likely secondary to venous congestion and compromised perfusion during the herniation episode. Neither at the time of the initial thoracotomy nor the subsequent laparotomies did we find any evidence of iatrogenic injury from opening the chest via the described methods. Given that we were at the bedside and unprepared to perform a thoracotomy, we made it a point to open the skin, subcutaneous fat, and muscle down to the pleural in between two ribs, then pop the pleura with a clamp and open the rest of it via blunt dissection with two fingers. In our case, this maneuver did not lead to iatrogenic defects of either the bowel or the lung, and there was no relevant bleeding. Due to the time pressure, we skipped our standard protocol for creating a sterile field but used only quick disinfection prior to incision and placed the eviscerated bowel onto a sterile towel. We later applied antibiotics accordingly and did not encounter significant infection of any of the parts involved.

The subsequent development of a chylothorax further illustrates the multifactorial morbidity in these patients, potentially resulting from lymphatic disruption or elevated central venous pressures.

Remarkably, despite prolonged shock and two resuscitations, the infant showed no evidence of hypoxic‐ischemic injury on MRI. Bilateral IVH was diagnosed (Grade II on the left and Grade II on the right). IVH refers to bleeding into the ventricular system, most commonly in premature infants. The severity is classified into four grades: Grade I (germinal matrix hemorrhage), Grade II (blood within the ventricles without dilation), Grade III (ventricular dilation due to blood), and Grade IV (extension into periventricular parenchyma). Grades I–II generally carry a low risk of long‐term neurological impairment, which is consistent with our clinical course. Postoperative recovery was uneventful, and neurological, cardiopulmonary, and nutritional function was restored by the time of discharge.

This case emphasizes several key lessons: (1) Late‐presenting CDH can cause sudden life‐threatening deterioration. (2) Tension enterothorax, although rare, should be considered in any neonate with mediastinal shift and shock without pneumothorax. (3) Emergency thoracotomy, even outside the operating room, as described in this case, can be a lifesaving last resort. (4) Multidisciplinary coordination and rapid surgical readiness are crucial. Our experience supports the consideration of bedside thoracotomy in extreme neonatal cases of obstructive cardiopulmonary collapse when caused by an unrelieved incarcerated thoracic lesion such as enterothorax.

## 4. Conclusion

Acute cardiopulmonary collapse caused by incarcerated CDH in neonates is rare but life‐threatening. In such extreme scenarios, emergency bedside thoracotomy, though highly uncommon in neonatal care, can serve as a decisive, lifesaving intervention, underscoring the importance of early recognition, rapid surgical decision‐making, and interdisciplinary preparedness to optimize outcomes.

## Funding

The authors received no specific funding for this work.

Open Access funding enabled and organized by Projekt DEAL.

## Ethics Statement

Written informed consent for publication and image use was obtained from the patient’s legal guardians.

## Conflicts of Interest

The authors declare no conflicts of interest.

## Data Availability

The data that support the findings of this study are available from the corresponding author upon reasonable request.
